# Economic Burden of Injuries argues for more Safe Communities in Iran and EMRO

**Published:** 2019-08

**Authors:** Koustuv Dalal

**Affiliations:** ^*a*^Senior Advisor, International Safe Community Certifying Centre (ISCCC) Stockholm, Sweden.

## Abstract

Globally, injuries cost nearly 1 trillion USD during 2016 equivalent to one-third of the total GDP EMRO countries. World Health Organization (WHO) and other United Nations (UN) agencies are reiterating policy advocacies and planning for injury prevention activities. Total burden of injuries during 2018 for the EMRO countries was 163 538 795 009 USD while only RTI burden was 46 056 411 787 USD (28.2%).

The EMRO countries are significantly loosing a big percentage of their GDPs due to amenable injury burden: Afghanistan 9.2%, Bahrain 1.93%, Djibouti 6.2%, Egypt 3%, Iran (Islamic Republic of) 4.3%, Iraq 12.95%, Jordan 3.24%, Kuwait 2.88%, Lebanon 3.81%, Libya 7.95%, Morocco 2.73%, Oman 3.46%, Pakistan 5.25%, Qatar 3.34%, Saudi Arabia 4.1%, Somalia 9.2%, Sudan 7.4%, Syrian Arab Republic 24.1%, Tunisia 2.98%, United Arab Emirates 2.81% and Yemen 10.3%.

Road Traffic Injuries constitute almost one-third of the total injury burden in the EMRO countries accruing mentionable amount of the countries’ GDPs: Afghanistan 1.2%, Bahrain 0.6%, Djibouti 1.9%, Egypt 0.9% Iran (Islamic Republic of) 1.96%, Iraq 1.3%, Jordan 1.7%, Kuwait 1.2%, Lebanon 0.96%, Libya 1.6%, Morocco 1.23%, Oman 1.72%, Pakistan 1.2%, Qatar 0.99%, Saudi Arabia 1.74%, Somalia 2.16%, Sudan 1.92%, Syrian Arab Republic 0.91%, Tunisia 1.33%, United Arab Emirates 0.85% and Yemen 1.92%.

In EMRO, the economic burden of injuries as percentage of GDP is engulfing almost the whole health expenditure (% of GDP) of the countries. In some countries it is more than double. Therefore due to injuries the EMRO countries are lacking high behind their healthcare budget resulting overall shortage of healthcare delivery.

Safe communities rely on interactive approach within the context of a partnership focusing sustainable health and welfare development through injury prevention and safety promotion. Most importantly it has emphasized on the policies and practices of governance and of collaboration in community development. Safe Communities are more cost-effective program than prevalence studies. Safe Communities are more successful as cost-effective policy tools rather than simple epidemiological studies.

Safe Communities have an estimated cost-benefit ratio of 1:10. When the countries are losing so much of their GDPs due to preventable injuries why not EMRO calls for more Safe Communities?

**Keywords:**

Economic Burden, Injury, Safe Community, Iran, EMRO

